# Case Report: Reinterpretation and Reclassification of *ARSB*:p.Arg159Cys Variant Identified in an Emirati Patient With Hearing Loss Caused by a Pathogenic Variant in the *CDH23* Gene

**DOI:** 10.3389/fped.2021.803732

**Published:** 2022-02-03

**Authors:** Nahid Al Dhahouri, Amanat Ali, Jozef Hertecant, Fatma Al-Jasmi

**Affiliations:** ^1^Department of Genetics and Genomics, College of Medicine and Health Sciences, United Arab Emirates University, Al-Ain, United Arab Emirates; ^2^Department of Pediatrics, Tawam Hospital, Al-Ain, United Arab Emirates

**Keywords:** arylsulfatase B, glycosaminoglycans, non-syndromic hearing loss, Cdh23, whole exome sequencing

## Abstract

Arylsulfatase B is an enzyme present in the lysosomes that involves in the breakdown of large sugar molecules known as glycosaminoglycans (GAGs). Arylsulfatase B chemically modifies two GAGs, namely, dermatan sulfate and chondroitin sulfate, by removing the sulfate group. Mutations in the gene encoding the arylsulfataseB enzyme causes lysosomal storage disorder, mucopolysaccharidosis type VI (MPS VI), or Maroteaux–Lamy syndrome. In this study, we report a case of congenital hearing loss with mild pigmentary changes in the retina, indicative of Usher syndrome, and a missense variant reported as likely pathogenic for MPS VI. Sequencing results identified a pathogenic missense variant p.Arg1746Gln in the *CDH23* gene. However, another missense variant *ARSB*:p.Arg159Cys was reported as likely pathogenic to the treating physician. Mutations in *ARSB* gene have been associated with MPS VI. Subsequently, ARSB enzyme activity was found low twice in dried blood spot (DBS), suggestive of MPS VI. The patient did not have the clinical features of MPS VI, but considering the wide clinical spectrum, progressive nature of MPS VI, and the fact that a treatment for MPS VI is available to prevent disease progression, further biochemical, enzymatic, and *in silico* studies were performed to confirm the pathogenicity of this variant. *In silico* tools predicted this variant to be pathogenic. However, the results of urine and serum GAGs and ARSB enzyme levels measured from patient's fibroblast were found normal. Based on clinical and biochemical findings, *ARSB*:p.Arg159Cys is likely benign and did not support the diagnosis of MPS VI. However, *CDH23*:p.Arg1746Gln, a pathogenic variant, supports the underlying cause of hearing loss. This study highlights the importance of a robust correlation between genetic results and clinical presentation, and biochemical and enzymatic studies, to achieve a differential diagnosis.

## Introduction

Mucopolysaccharidoses (MPSs) are lysosomal storage disorders (LSDs) caused by inherited deficiencies of lysosomal hydrolases responsible for the catabolism of mucopolysaccharide molecules known as glycosaminoglycans (GAGs) (1). To date, 13 enzyme deficiencies have been identified that lead to an accumulation of different species of GAGs including dermatan sulfate (DS), heparan sulfate (HS), keratan sulfate (KS), chondroitin sulfate (CS), and hyaluronic acid in blood, tissues, and urine (2). MPSs are autosomal recessive disorders, except for MPS type II also known as Hunter syndrome, which is an X-linked disorder (3). MPS VI have a variable clinical phenotype in terms of severity and progression of disease, with multisystemic involvement that can include skeletal deformities, coarse facial features, and organomegaly. Hearing loss is one of the most common clinical presentation in MPSs (4). Studies have reported confirmed cases of conductive hearing loss, sensorineural hearing, and mixed hearing loss in MPS VI (5). However, patients with MPSs usually do not present with congenital hearing loss. Therefore, an intensive clinical evaluation is crucial to establish a differential diagnosis. MPS VI, or Maroteaux–Lamy syndrome, is caused by a deficiency of N-acetylgalactosamine-4-sulfatase deficiency (arylsulfatase B), leading to an accumulation of DS (OMIM: 253200). In newborns, the incidence of MPS VI is 1 in 250,000–600,000. However, the prevalence was observed to be higher in consanguineous populations (6, 7). Primary treatment options for MPSs include hematopoietic stem cell transplantation (HSCT) and enzyme replacement therapy (ERT) (8). ERT in MPS VI has been shown to reduce GAG accumulation in clinical trials (9). However, the effects of ERT on the hearing loss is inconclusive (10). Differential diagnosis based solely on clinical observations are rarely conclusive, and therefore, additional biochemical and genetic confirmatory tests are often required. Liquid chromatography tandem mass spectrometry (LC-MS/MS) is an effective method for the measurement of metabolites such as disaccharides derived from glycosaminoglycans (11). The CDH23 is a long protein made up of 27 extracellular cadherin (EC) repeats and a non-canonical domain. Each EC repeat is composed of approximately 110 residues. Linker regions that connect different EC repeats are highly conserved and are important for the binding of Ca^2+^ ions (12).

In this study, we report a pathogenic variant *CDH23*:p.Arg1746Gln in a case of congenital hearing loss with mild pigmentary changes in the retina, indicative of Usher syndrome, and a missense variant reported as likely pathogenic for MPS VI identified through whole exome sequencing (WES). However, biochemical confirmatory results for MPS VI were found negative. This report highlights the importance to identify, classify, and characterize variants using computational, biochemical, and clinical approaches.

## Materials and Methods

### Ethical Approval

The Ethical Approval was obtained from Abu Dhabi Health Research and Technology Committee, reference number (DOH/CVDC/2020/1185). The affected case was diagnosed with hearing loss at birth and referred to Tawam Hospital, Abu Dhabi, for further clinical evaluation. Different biological samples such as blood, urine, and fibroblasts were collected to conduct several assays as part of the clinical investigation. Patient was consented for WES, and after that, his blood was spotted on CentoCard (Centogene AG, Germany).

### Biochemical Analysis

#### Arylsulfatase B Fluorimetry Assay

The enzymatic assay is a screening method for MPS VI based on the quantitative determination of ARSB (aryl-sulfatase B) activity in dried blood spots (DBS). The patient's blood was spotted on CentoCard, left to dry for 24 h, and was shipped to Centogene AG (Rostock, Germany). The protocol involves extraction of the ARSB from the DBS, incubation with a specific synthetic substrate for 20 h, and detection of the enzymatic product using fluorimetry. Quantification was performed on a fluorimeter using an external calibration line of the enzymatic product. Affected MPS VI patients show low activity of ARSB, while the healthy controls show high activity of the same enzyme (13).

#### Dimethylene Blue Assay

Total urine GAGs were measured using the dimethylene blue (DMB) assay, which involves binding of GAGs to the dye DMB followed by a spectrophotometric analysis of the GAG-DMB complex (14).

#### Mucopolysaccharidosis Quant S

The patient's serum sample was shipped to Mayo Clinic (FL, USA) for quantification of glycosaminoglycans (GAGs). The selected reaction monitoring (SRM) mode in liquid chromatography–electrospray ionization–tandem mass spectrometry (LC–ESI–MS/MS) was used for quantitative measurement of GAGs. The simultaneous quantification of 23 differently sulfated disaccharides from four GAG classes (8 chondroitin/dermatan sulfates, 1 hyaluronic acid, 12 heparan sulfates, and 2 keratan sulfates) was achieved using MS/MS technique. Apart from the internal disaccharides as mentioned previously, some saccharides derived from the non-reducing terminal were also observed. The chain length of GAGs was estimated based on the simultaneous quantification of both internal and non-reducing terminal saccharides (15).

#### Arylsulfatase B Enzymatic Assay

Enzymatic assay was performed on the fibroblast cell culture obtained from the index skin biopsy. Enzyme assay is the gold standard for diagnosis of patients with MPS VI. It can also determine the pathogenicity effect of the detected novel variants. Skin biopsy was shipped to South Australia Pathology Laboratory (Adelaide, Australia). In normal physiological conditions, the ARSB enzyme chemically modifies chondroitin-4-sulfate and dermatan sulfate by removing the sulfate group. The kinetic parameters, Michaelis–Menten constant (Km), and maximum velocity of human ARSB activity in cultured skin fibroblasts were measured with a different synthetic substrate similar to chondroitin 4-sulfate and dermatan sulfate. The natural substrate's aglycon structures were desulfated up to 4,400 times faster than the substrates specific for arylsulfatase-B (N-acetylgalactosamine 4-sulfate). The enzyme activity of arylsulfatase B in skin fibroblast was measured using O-(beta-N-acetylgalactosamine 4-sulfate)-(1–−4)-O-D-(beta-glucuronic acid)-(1–−3)-O-D-N-acetyl[1-3H] galactosaminitol 4-sulfate. These substrates are used to identify Maroteaux–Lamy syndrome patients from normal controls or/and MPSs patients (16).

### DNA Extraction and Whole Exome Sequencing

The patient's blood was spotted on CentoCard and left to dry for 24 h. DNA extraction was performed on QIAcube instrument with QIAamp DNA Blood Mini QIAcube Kit (Qiagen, Valencia, CA, USA) following the manufacturer's instructions. NanoQuant plate was used for the quality and quantity analysis of DNA samples. The diagnostic WES was carried out by Centogene AG (Rostock, Germany). Briefly, twist Human Core Exome Plus kit was used for the exome capture, and the captured libraries were target enriched and indexed. A Novaseq 6000 sequencer was used to sequence the target enriched libraries. For the WES data analysis, sequencing reads were initially converted into normal FastQ format and were processed via locally maintained bioinformatics pipeline. The short reads were aligned to the GRCh37 (hg19) build of the human reference genome. Aligned sequences were further converted to a binary BAM file format, followed by variant calling, which was performed on the secondary alignment files using GATK Haplotype Caller, free bayes, and sam tools. Locally maintained bioinformatics tools and Annovar were used for variants annotation. Alignments were conducted and visually confirmed by using Alamut v.2.4.5. The identified variants were further analyzed for genotype phenotype correlation according to the guidelines of the American College of Medical Genetics (ACMG).

### *In silico* Analysis

To further examine the deleterious effect of the identified missense variants on CDH23, and ARSB, several *in silico* tools, namely, Sorting Intolerant from Tolerant (SIFT) (17), Polymorphism Phenotyping v2 (PolyPhen-2.0) (18), Fathmm (19), and Mutation Taster (20), were used. Multiple sequence alignment (MSA) was performed to determine the conservation of amino acids at the missense position (p. Arg159Cys) in ARSB and (p.Arg1746Gln) in CDH23, and Jensen–Shannon Divergence (JSD) scores were also calculated (21). Amino acid sequences of CDH23, and ARSB protein from *Homo sapiens* (human), *Mus musculus* (house mouse), *Rattus norvegicus* (Norway rat), *Callorhinchus milii* (elephant shark), *Bos taurus* (cattle), *Danio rerio* (zebrafish), *Felis catus* (domestic cat), *Pan troglodytes* (chimpanzee), and *Vulpes Vulpes* (red fox) were retrieved from National Center for Biotechnology Information (NCBI) RefSeq and aligned using Clustal Omega (22). JSD scores were calculated using aligned sequences in FASTA format. The protein sequence of the human CDH23 (accession number: Q9H251) and ARSB (accession number: P15484) were obtained from UniProt for homology modeling. Protein databank (PDB) IDs 1FSU and 5VVM were used as templates to produce the homology models of wild-type and mutant CDH23 and ARSB, respectively, using SWISS-MODEL. PyMOL was used for the evaluation and visualization of generated models (23).

## Results

### Case Report Summary

**Figure 1:**
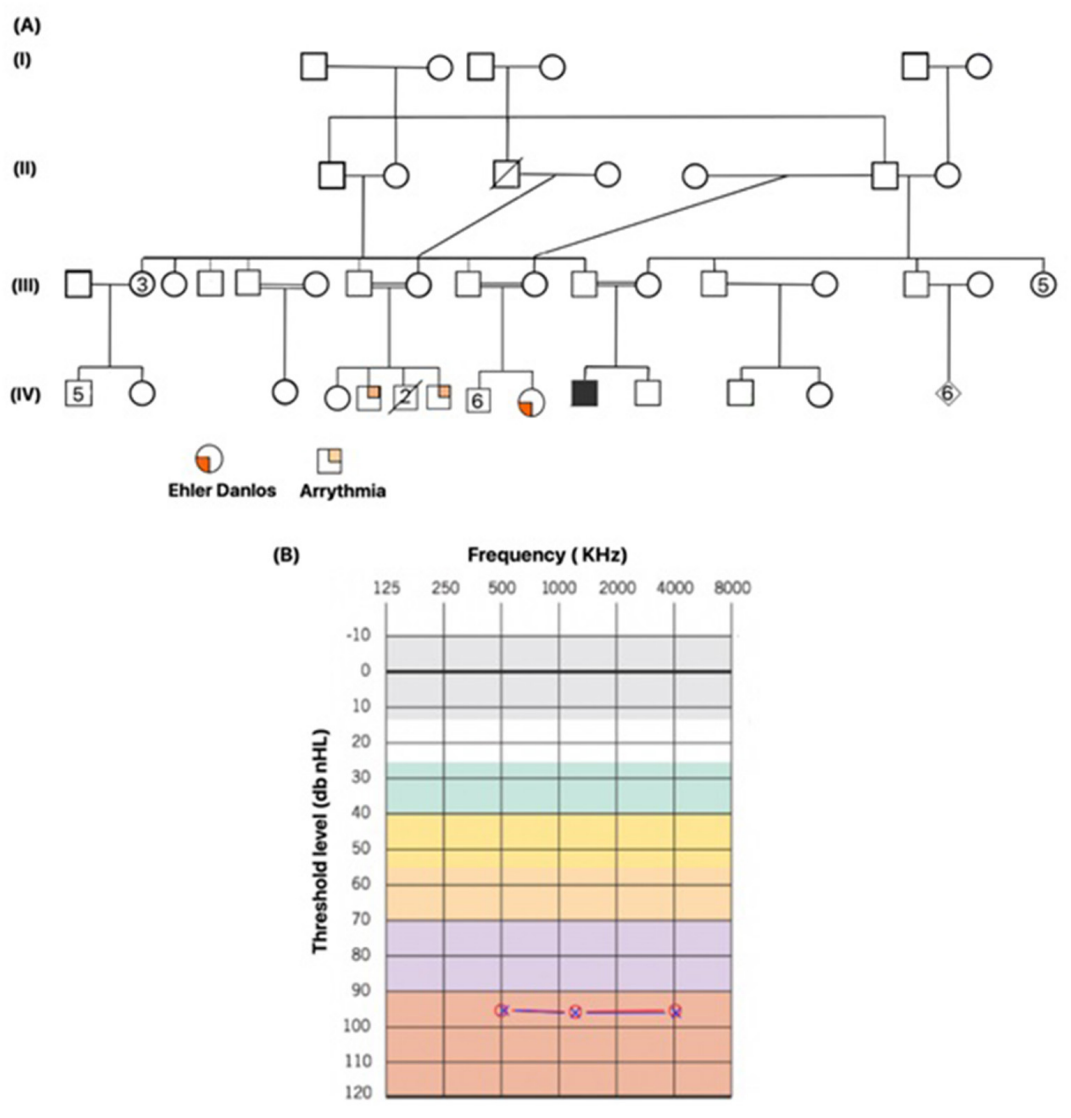
The affected patient's family pedigree and the results of a confirmatory hearing loss test. **(A)** Family pedigree of the index patient, showing a highly inbred family. Family has a history of Ehler Danlos syndrome and history of cardiac arrythmia. The index patient is indicated with filled square. Male and female are shown in squares and circles, respectively. **(B)** Threshold test indicating the level of hearing loss recorded from the auditory brainstem response (ABR), classifying it to be severe. Background colors represent the severity of hearing loss. Normal hearing (gray and white), mild hearing loss (green), moderate hearing loss (yellow), moderately severe hearing loss (orange), severe hearing loss (purple), and profound hearing loss (red). Measurements were recorded in both ears at three frequencies. Red circle indicates the value recorded in left ear, blue cross in a red circle indicates the value recorded in right ear.

A male proband was born after full-term pregnancy *via* spontaneous vaginal delivery (SVD) to consanguineous Emirati parents. During newborn screening, hearing loss measurements were performed using distortion product oto-acoustic emission with the interacoustic instrument at 30–40 db prior to the infant's discharge. The proband failed the test at 40 db and was suggestive of congenital hearing loss. A diagnostic auditory brainstem response (ABR) was performed to measure the degree of hearing loss, and threshold estimation was computed during normal sleep. ABR results revealed no reproducible wave V traced out at 90 dB nHL for click stimulus and tone burst (0.5, 1.0, and 2.0 kHz) in both ears, indicative of bilateral hearing loss. Cochlear microphonics was absent. Moreover, two additional audiology tests performed every 2 months showed no repeatable and reliable response even at 90dB nHL for clicks at 0.5, 1.0, 2.0, and 4 kHz tone burst in both ears, confirming a profound sensorineural hearing loss (Figure 1B). Patient underwent an ear tube surgery at 8 months of age followed by a cochlear implant at the age of 10 months. Mild pigmentary changes in the retina were observed during an eye examination, suggestive of Usher syndrome. To investigate the genetic causes of congenital hearing loss, gene panel for hearing loss at Prevention Genetics (Marshfield, WI, USA) was ordered, and results came negative. Additionally, the results of microarray were also found to be negative when compared to a normal male array. The patient is from a highly consanguineous family, and his parents are first cousins. The proband also had a family history of Ehlers–Danol syndrome (EDS) and sudden cardiac death for several family members (Figure 1A). Whole exome sequencing was ordered to determine the genetic reasoning of hearing loss. Sequencing results identified a missense variant *CDH23*:p.Arg1746Gln in a homozygous state associated with hearing loss. *In silico* tools predicted this as pathogenic. ClinVar ACMG classifies it as pathogenic. Furthermore, an incidental finding of likely pathogenic mutation *ARSB*:p.Arg159Cys based on Centogene ACMG criteria, suggestive of MPS VI diagnosis, was also reported to the treating physician. As per Centogene local database, this variant has been previously identified in one second affected patient in a homozygous state with partial phenotype overlap with no enzymatic analysis. ARSB enzyme activity (EA) measured twice in DBS revealed low levels of EA. Based on this, *ARSB*:p.Arg159Cys variant was reported as likely pathogenic. At that time, this variant was not reported in any public database. However, this variant has recently been classified as variant of unknown significance (VUS) in ClinVar. MPS VI is a disease that progresses slowly or quickly, but early intervention is critical for the patient's outcome. The proband had a history of inguinal hernia repair, recurrent ear infection, recurrent fever, and reactive airway disease. Patient's developmental milestones were normal, rolling over 40 days, sitting at 6/12 months, standing at 1.5 years. Gross motor and fine motor skills were also observed normal. Moreover, he received a speech therapy three times per week. Physical examination performed at 30 months of age showed head circumference of 47 cm (5th percentile), height of 92 cm (25th percentile), weight of 13 kg (25th percentile), no corneal clouding, no coarse fascial feature, no organomegaly, and no joint restriction. Furthermore, skeletal survey did not show any evidence of dysostosis multiplex; ultrasound of abdomen was observed normal with no hepatosplenomegaly. Echo of the heart was also normal. The patient did not have the clinical features of MPS VI, but considering the wide clinical spectrum, progressive nature of MPS VI, and the fact that a treatment for MPS VI is available to prevent disease progression, further *in silico*, biochemical, and enzymatic studies were performed to confirm the pathogenicity of *ARSB*:p.Arg159Cys variant. *In silico* tools predicted this variant to be pathogenic. However, the results of urine and serum GAGs came normal. ARSB enzyme levels measured from patient's fibroblast were also found normal. These studies conclusively ruled out the diagnosis of MPS VI.

### Molecular and Biochemical Studies

WES was performed to determine the genetic basis of hearing loss. Variants were prioritized on the basis of clinical phenotypes of the patient, family history, suspected disease pathways, and minor allele frequency (≤0.05). Sequencing results identified a homozygous missense variant *CDH23*:p.Arg1746Gln. It has already been reported in ClinVar as pathogenic. This confirmed the diagnosis of hearing loss (Table 1). Additionally, a missense variant *ARSB*:p.Arg159Cys was found during WES analysis in a homozygous state. At the time of identification, this variant was not reported in any public database. Moreover, variant around this region in ARSB has been found associated with MPS VI (24). ARSB enzyme was estimated in DBS, and results showed low levels of ARSB enzyme 6.3 μmol/L/h (reference, >8.8 μmol/L/h), supporting the diagnosis of MPS VI. Since it is a screening assay and has high false-positive rate, further biochemical and enzymatic studies were performed. Urine GAGs assay was performed as part of the biochemical clinical investigation, and the result was normal at 18.8 mg/mmol creatinine (reference value, <24.0 mg/mmol creatinine). MPS serum assay was carried out, and results indicated the GAGs levels including dermatan sulfate, heparin sulfate, and total Keratan sulfate to be normal (Table 2). Furthermore, an enzymatic assay was performed on the patient's skin fibroblast to determine the deficiency in ARSB enzyme. However, ARSB enzyme levels were found to be normal at 12.0 pmol/min/mg protein (reference range, 11.8–39.4 pmol/min/mg/protein). This variant has recently been deposited in ClinVar database and classified as VUS based on ClinVar ACMG classification. However, our results conclusively indicated the likely benign nature of *ARSB*:p.Arg159Cys variant.

**Table 1:** Description of variants detected during whole exome analysis.

**Gene**	**Variant coordinates**	**Amino acid change**	**Zygosity**	**Type**	**Clinvar ACMG Classification**
*CDH23*	NM_022124.5:c.5237G>A	p.(Arg1746Gln)	Homozygous	Missense	Pathogenic
*ARSB*	NM_000046.3:c.475C < T	p.(Arg159Cys)	Homozygous	Missense	Variant of unknown significance

**Table 2:** Results of mucopolysaccharides quant assay performed on patient's serum.

**Mucopolysaccharides quant, S**	**Concentration (ng/ml)**	**Reference value**
Dermatan sulfate	61.16	<300
Heparan sulfate	16.52	<55
Total keratan sulfate	1,078.87	<1,800

### *In silico* Analysis

**Figure 2:**
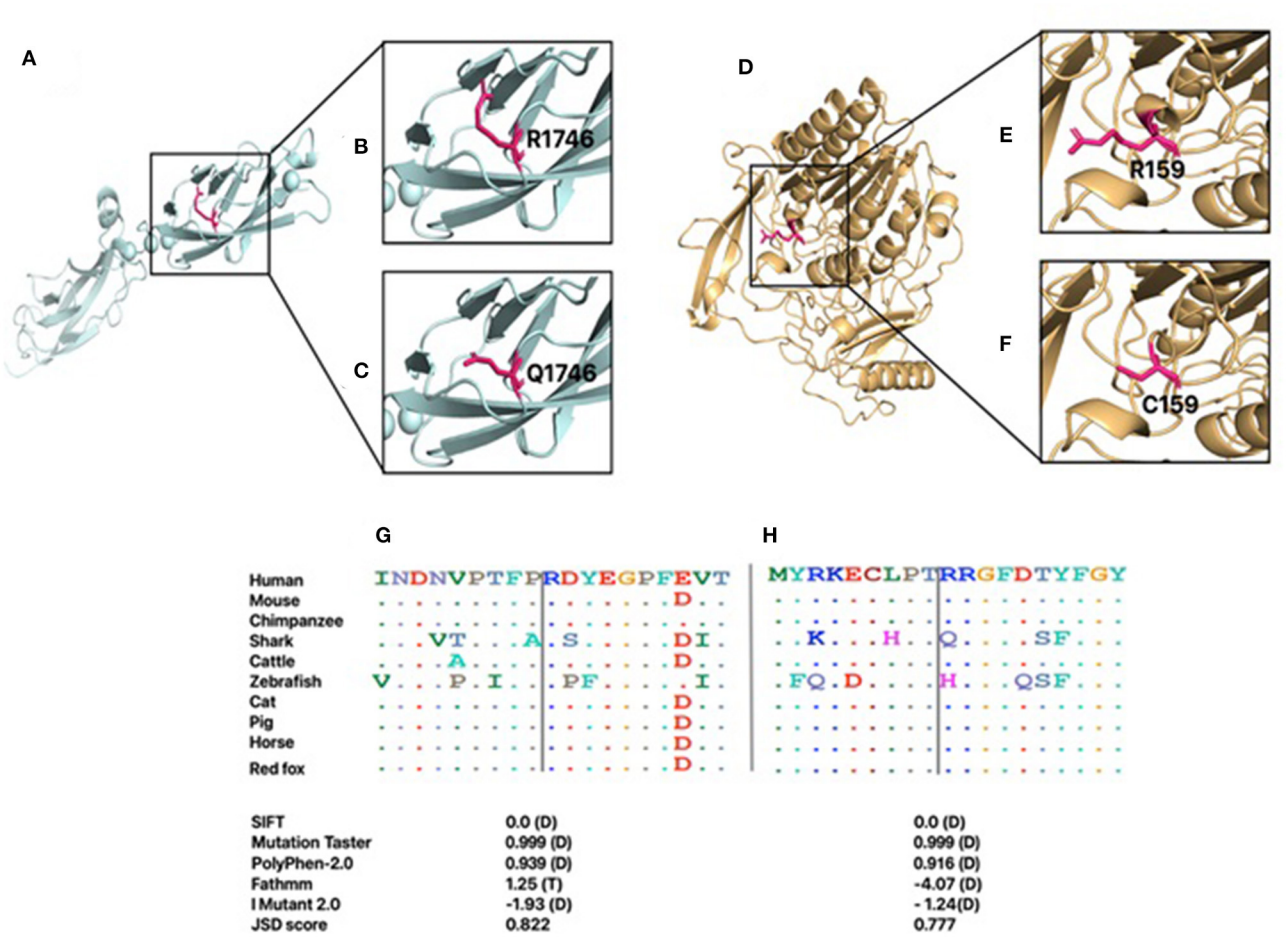
The generated homology models for CDH23 and ARSB. The proteins are shown in cartoon representation, and the amino acid is demonstrated with a stick representation. The boxed region in A and D is enlarged in subsequent images. **(A)** Modeled structure of CDH23. **(B)** Wild-type R1746. **(C)** Mutant Q1746. **(D)** Modeled structure of ARSB. **(E)** Wild-type R159. **(F)** Mutant C159. Conservation of each amino acid is shown across species for both CDH23 **(G)** and ARSB **(H)**. Predicted effects of the variants on protein structure and function based on SIFT, Fathmm, Mutation Taster, PolyPhen2.0, and I Mutant2.0 programs. D, damaging; T, tolerated.

Several *in silico* variant predictions tools and molecular modeling were utilized to assess the variant pathogenicity effect on the overall protein structure, stability, and function. CDH23 and ARSB protein sequences of different mammals were retrieved from NCBI's reference sequence (RefSeq), and MSA was carried out using Clustal Omega (22). Arg1746 and Arg159 residues of CDH23 and ARSB are highly conserved as indicated by their JSD scores (Figures 2G,H). *In silico* tools consistently predicted these variants to be pathogenic. I-Mutant tool was used to compute the impact of *CDH23*:p.Arg1746Gln and *ARSB*:p.Arg159Cys variants on the overall stability of the protein. Both variants decreased the protein stability as indicated by their Gibbs free energy change value (ΔΔG) (Figures 2G,H). Importantly, the results of *in silico* tools for the *ARSB*:p.Arg159Cys variant were found to be contradictory to its functional studies. Moreover, three-dimensional models of wild-type and variants of ARSB and CDH23 were generated using SWISS-MODEL to observe the effect of missense variants on the protein structure and function. Initially, the full amino acid sequence of human CDH23 was used for the identification of appropriate templates. However, no good quality template was found. In order to obtain a reasonable template, only EC17 and EC18 residues of human CDH23 were selected for template search. Human CDH23 (EC17 and EC18) produced 90% sequence identity with three-dimensional structure of mouse CDH23 (EC17 and EC18), indicating a good template for modeling. Arg1746Gln variant is located in EC17 of CDH23 (Figure 2A). The substitution of a charged residue arginine to glutamine is physiochemically significant (Figures 2B,C). Arg1746 forms a hydrogen bond with Glu1749. The substituted Gln1746 is likely to disrupt this interaction. It is also perceivable that this loss of charge mutation could likely affect salt bridges and hydrogen bonds that are essential in the CDH23 cis homodimer interface.

The 3D structure of human ARSB is available in PDB (PDB ID: 1FSU) and was used as a template for generating the 3D model of *ARSB*:p.Arg159Cys variant (Figure 2D). Arg159 is located in a loop region of ARSB (Figures 2E,F). This region is considered the probable recognition site for targeting to the lysosomes (25). The substitution of arginine to cystine is physiochemical significant. However, Arg159 is not the part of residues that line the active site pocket of ARSB, which is essential for enzyme activity. Aside from the loss of a charged residue at the surface of enzyme, no notable impact is expected.

## Discussion

This study presents a pathogenic variant p.Arg1746Gln in *CDH23* gene associated with congenital hearing loss with mild pigmentary changes in the retina, indicative of Usher syndrome, and an incidental finding of a missense variant (*ARSB*:p.Arg159Cys) reported as likely pathogenic for MPS VI in an Emirati patient.

A series of biochemical and enzymatic assays were performed to establish the pathogenicity of *ARSB*:p.Arg159Cys associated with MPS VI. The normal results of enzyme activity on fibroblast and normal level of GAGs indicate that this variant is likely benign. This study also highlights the limitations of *in silico* tools that consistently predicted this variant to be pathogenic. Therefore, a careful evaluation of clinical phenotypes and functional studies is imperative to confirm variant pathogenicity.

It is critical to establish a clear genotype–phenotype correlation early in the diagnostic process. Generally, a combination of clinical and laboratory findings is needed to establish the diagnosis of MPSs. The patients of MPS VI often present with skeletal deformities, coarse facial features, conductive hearing loss, and corneal clouding. It is worth mentioning that the physical examination of index patient was inconsistent with the clinical signs and symptoms of MPS VI. Despite a clear physical examination, the patient's history of inguinal hernia repair, the detection of *ARSB*:p.Arg159Cys variant, and positive results of ARSB enzyme activity in DBS, this patient was suspected with MPS VI. It is also worth mentioning that the variant present close to this region in ARSB is reported to be associated with MPS VI (1, 26).

Interestingly, the gene panel performed in the first place failed to identify any variant associated with hearing loss. There are several limitations associated with the use of gene panel assays, such as variants are limited to pre-selected gene. Additionally, it requires regular updates, as new variants are being discovered (27). It is also important to mention that *CDH23*, a disease-causing gene linked with hearing loss was not included in the design of gene panel. This is why the gene panel failed to identify any mutation in genes linked to hearing loss. However, WES results identified a pathogenic variant *CDH23*:p.Arg1746Gln associated with hearing loss.

Finding likely pathogenic variants necessitates immediate action in patients' management plan, especially if a treatment is available. According to ACMG/AMP guidelines, pathogenic and likely pathogenic variants are reported with 99.1 and 90% confidence (28). However, physicians generally interpret both pathogenic and likely pathogenic variants to be disease associated. According to a recent study that evaluated 36,808 variants classified as likely pathogenic in ClinVar, only 2.16% were reclassified to other categories (29). Interestingly, only four variants were reclassified from likely pathogenic to likely benign (29). This suggests that likely pathogenic variants are rarely found to be benign. Therefore, exhaustive interpretation of genetic data should be performed, before reporting genetic results to the treating physicians.

Here, sequencing results initially reported a likely pathogenic variant *ARSB*:p.Arg159Cys. *ARSB* gene variants have often been associated to the lysosomal storage disorder MPS VI (30). Conductive hearing loss has been linked to MPS VI and other MPS subtypes (31). ARSB enzyme levels in the patient's DBS were measured twice, and the results supported the MPS VI diagnosis. It is worth highlighting that this assay is a screening assay and has high false-positive rate. Therefore, additional biochemical assays should be performed to confirm the diagnosis of MPS VI due to its experimental limitations. Measuring ARSB in DBS is performed using a fluorescence-based method that is being used for detecting LSD in newborn screening (NBS), where different β-methylumbelliferone sulfate (β-MU)-derived substrates similar to natural substrates are applied to quantify lysosomal enzyme deficiency in DBS samples (32). β-MU molecule is known to be highly fluorescent; its detection is often hindered when it is found in a DBS (13). Normally, known standards of β-MU are prepared in an aqueous matrix lacking blood components. Therefore, the enzyme activity (EA) calculated by interpolating sample fluorescence values in a blood-free calibration curve provides data that underestimate the real EA. This could potentially explain the reason why enzyme ARSB on DBS was found lower twice (32). Therefore, enzymatic screenings performed in DBS are not always conclusive, and it required further confirmatory biochemical assays. Additional biochemical and enzymatic studies were performed to establish the pathogenicity of *ARSB*:p.Arg159Cys; however, results were found normal (Table 2). These findings concluded that the enzyme is functional, and thus, the patient cannot be diagnosed with MPS VI.

As the number of genetics and genomics tests are increasing, interpreting sequence variants becomes more difficult. Recently, several sequence variants of VUS have been identified (31). Although ACMG guidelines provides a robust framework for laboratories to determine the pathogenicity of sequence variants in a systematic manner, the degree of uncertainty provided by these guidelines can produce contrary classification among different clinical laboratories (32). A study performed on variants reported in ClinVar observed a conflicting interpretation rate of 11.7% among four clinical laboratories (33). Moreover, variability in the *in silico* tools and classification of functionally relevant variants also pose a challenge on the interpretation of variants. In clinical settings, predictions from *in silico* algorithms are considered as one of the eight evidence criteria recommended for variant interpretation by ACMG guidelines (34). *In silico* tools often produce high rate of false-negative and false-positive results. In our study, multiple *in silico* tools consistently predicted *ARSB*:p.Arg159Cys variant as pathogenic, contradicting functional studies that found the variant to be likely benign. Therefore, the patient does not require immediate enzyme replacement therapy.

## Conclusion

In this study, we report the utility of WES in identifying the genetic variants associated with clinical phenotypes. A robust genotype–phenotype correlation and functional studies are needed to establish the pathogenicity of detected variants during differential diagnosis. Our biochemicals and enzymatic studies indicate that *ARSB*:p.Arg159Cys variant is likely benign and should be reclassified from VUS to likely benign.

## Data Availability Statement

The original contributions presented in the study are included in the article/supplementary materials, further inquiries can be directed to the corresponding author.

## Ethics Statement

The studies involving human participants were reviewed and approved by Abu Dhabi Health Research and Technology Committee, Department of Health, Abu Dhabi, United Arab Emirates. Written informed consent to participate in this study was provided by the participants' legal guardian/next of kin.

## Author Contributions

FA-J conceived and managed the funding of this study. NA, AA, and FA-J designed the study. Material preparation and molecular analysis were performed by NA, AA, and FA-J. Patient examination and clinical analysis were performed by FA-J, and JH. NA, and AA wrote the manuscript. All authors have read and agreed to the published version of the manuscript.

## Funding

The research project was funded by the United Arab Emirates University grant (31M491) to FA-J. The funder has no role in study design, data collection, and decision to publish.

## Conflict of Interest

The authors declare that the research was conducted in the absence of any commercial or financial relationships that could be construed as a potential conflict of interest.

## Publisher's Note

All claims expressed in this article are solely those of the authors and do not necessarily represent those of their affiliated organizations, or those of the publisher, the editors and the reviewers. Any product that may be evaluated in this article, or claim that may be made by its manufacturer, is not guaranteed or endorsed by the publisher.
